# Topographical Organization of Attentional, Social, and Memory Processes in the Human Temporoparietal Cortex^1^,^2^,^3^

**DOI:** 10.1523/ENEURO.0060-16.2016

**Published:** 2016-04-29

**Authors:** Kajsa M. Igelström, Taylor W. Webb, Yin T. Kelly, Michael S. A. Graziano

**Affiliations:** Princeton Neuroscience Institute and Department of Psychology, Princeton University, Princeton, New Jersey 08544

**Keywords:** angular gyrus, blind source separation, data-driven fMRI analysis, superior temporal gyrus and sulcus, supramarginal gyrus

## Abstract

The temporoparietal junction (TPJ) is activated in association with a large range of functions, including social cognition, episodic memory retrieval, and attentional reorienting. An ongoing debate is whether the TPJ performs an overarching, domain-general computation, or whether functions reside in domain-specific subdivisions. We scanned subjects with fMRI during five tasks known to activate the TPJ, probing social, attentional, and memory functions, and used data-driven parcellation (independent component analysis) to isolate task-related functional processes in the bilateral TPJ. We found that one dorsal component in the right TPJ, which was connected with the frontoparietal control network, was activated in all of the tasks. Other TPJ subregions were specific for attentional reorienting, oddball target detection, or social attribution of belief. The TPJ components that participated in attentional reorienting and oddball target detection appeared spatially separated, but both were connected with the ventral attention network. The TPJ component that participated in the theory-of-mind task was part of the default-mode network. Further, we found that the BOLD response in the domain-general dorsal component had a longer latency than responses in the domain-specific components, suggesting an involvement in distinct, perhaps postperceptual, computations. These findings suggest that the TPJ performs both domain-general and domain-specific computations that reside within spatially distinct functional components.

## Significance Statement

The temporoparietal junction (TPJ) is a major communication hub in the human brain. The exact pattern of overlap and separation of function in the TPJ has been difficult to study due to the complexity of its responses during many different kinds of tasks. We studied the activity in the TPJ during five behavioral tasks associated with attention, memory retrieval, and social cognition. We found that one zone in the TPJ was active in all five tasks, whereas other zones were active in a more task-specific manner. Our findings suggest that the TPJ is a site where multiple brain networks converge and interact, but that it also contains more functionally specific subregions.

## Introduction

Many theories have been proposed to explain the multitude of tasks that activate the temporoparietal junction (TPJ), which involve functions ranging from bottom-up attention to episodic memory retrieval and social cognition. For example, it has been suggested that the episodic memory activity in the TPJ is related to reflexive orienting to information retrieved from memory (Cabeza, 2008; Ciaramelli et al., 2008; Cabeza et al., 2011), and that social cognition in the TPJ might depend on similar low-level information processing (Decety and Lamm, 2007). Other theories have postulated that the TPJ is a zone of convergence and integration, in which internal models of one’s environment, social context, or attentional state are maintained and updated (Graziano and Kastner, 2011; Carter and Huettel, 2013; Geng and Vossel, 2013; Kelly et al., 2014; Webb and Graziano, 2015). Comparisons of activation patterns within single subjects have shown some separation but also zones of overlap. For example, topographic overlap has been reported between theory-of-mind and attentional reorienting activity (Mitchell, 2008); between memory retrieval and attentional reorienting (Cabeza et al., 2011); and among theory-of-mind, attentional reorienting, and biological motion (Lee and McCarthy, 2016). However, functional heterogeneity has also been seen in both meta-analyses and within-subject fMRI studies, manifesting as the physical separation of processes, an ability of multivoxel pattern analysis to discriminate different tasks within regions of overlap, and distinct connectivity patterns of activation foci (Hutchinson et al., 2009; Scholz et al., 2009; Cabeza et al., 2011, 2012; Daselaar et al., 2013; Lee and McCarthy, 2016).

The great spatial variability of fMRI activations in the TPJ is perhaps not surprising given the intersubject heterogeneity in the TPJ, the limitations of normalizing individual brains into a common space, and the limitations of voxelwise analysis. One useful way of addressing TPJ function is careful within-subject analysis of task-related activity in high-resolution fMRI scans (Mitchell, 2008; Scholz et al., 2009; Cabeza et al., 2011; Lee and McCarthy, 2016). Another possibility, explored in this study, is to use multivariate data-driven methods to work around the limitations of voxelwise analysis. We previously found that localized independent component analysis (local-ICA), which decomposes the fMRI signal in the TPJ into a linear mixture of spatiotemporal source processes, could be used to parcellate the TPJ into five to six subdivisions per hemisphere (Igelström et al., 2015, 2016). These components included bilateral posterior (TPJp), anterior (TPJa), dorsal (TPJd), and ventral (TPJv) regions, and a central right-biased region (TPJc). The time courses of the independent components (ICs) within the TPJ were correlated with distinct resting-state networks (Igelström et al., 2015, 2016), indicating that they represented functionally distinct processes. This method of local-ICA was robust across multiple independent subject cohorts (Igelström et al., 2015).

We hypothesized that local-ICA, by isolating functional processes from noise and by providing IC time courses that can be analyzed for task relatedness, may be a powerful approach to study the distribution of processes in the TPJ. A primary goal of this experiment was to test a diversity of tasks that in the previous literature have been shown to evoke robust activity in the TPJ. This diversity of tasks allowed us to ask basic questions, such as the following: is the TPJ heterogeneous, with subareas that tend to be recruited in different tasks, or is it a site of convergence, with a generalized activity that is similar across a range of tasks? Or does the TPJ have some combination of properties, with some subregions showing task-specific activity and other subregions showing functional overlap? To pursue that goal, five tasks were chosen based on their prominent roles in the TPJ literature, and their diversity in terms of behavioral paradigm and cognitive function, although these tasks did not constitute an exhaustive list of all tasks that may be relevant. The tasks included (1) a social attribution-of-belief task (Dodell-Feder et al., 2011), (2) an old/new episodic memory retrieval task (Konishi et al., 2000), (3) an attribution-of-attention task (Kelly et al., 2014), (4) a Posner attentional reorienting task (Mitchell, 2008), and (5) an oddball target detection task (Stevens et al., 2000). We analyzed the fMRI signal during these tasks by using local-ICA to decompose the signal within the temporoparietal cortex. Task-related ICs were identified using multiple regression analysis, and their network participation was mapped using functional connectivity analysis. This data-driven approach revealed a functional topography within the temporoparietal cortex that included zones of both task convergence and task specialization.

## Materials and Methods

**Figure 1.**
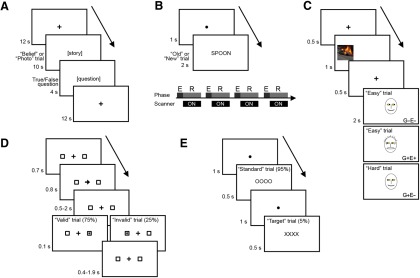
Schematic representation of task designs. ***A***, Theory-of-mind task. A story requiring either attribution of belief or reasoning about a photo was shown for 10 s, followed by a true/false question for 4 s. There were 20 trials in total. ***B***, Episodic memory retrieval task. The task was divided over four runs, each consisting of an encoding phase (“E”) before the run began, and a retrieval phase (“P”; bottom). In the encoding-phase, subjects were asked to memorize words presented sequentially on the screen (33 words). In the retrieval run (25 old words intermixed with 50 new words and 25 fixation trials), subjects indicated with a button press whether a word was old or new. There were 400 trials in total. ***C***, Attribution-of-attention task. An object with either negative or positive salience was presented on the right or left for 1 s (this example shows a car fire). After a 0.5 s interval, the face of a cartoon character was presented centrally for 2 s. Its gaze was directed either toward or away from the object (G+ or G−), and its emotional expression either matched or mismatched the valence of the object (E+ or E−). Subjects rated the character’s level of awareness of the object on a scale of 1 (not aware) to 3 (very aware). Four trial types were possible: G−E−, G+E+, G+E−, and G−E+. The trials in which the gaze and expression cues were inconsistent (G+E− and G−E+) were labeled as hard trials (for more details, see Materials and Methods). There were 384 trials in total. ***D***, Attentional reorienting task. A central cue pointing right or left predicted the location of a target in 75% of trials. Subjects were asked to indicate which side the target appeared on. There were 200 trials in total. ***E***, Target detection task. A visual standard stimulus (“OOOO”) was presented on the screen every 1.5 s. In 5% of trials, this was replaced by the target stimulus (“XXXX”). The subjects silently counted how many targets they saw. There were 480 trials in total.

The experimental approach consisted of four main steps. First, fMRI data were collected during behavioral tasks that in prior studies evoked activity in the TPJ (Fig. 1). Second, for each task, local-ICA was used on the group level to extract the dominant spatiotemporal TPJ processes in a data-driven manner. Third, the IC time courses from these task-specific ICAs were entered into mixed-effects multiple-regression analyses to identify components that were significantly task related. Fourth, all IC time courses were used for a functional connectivity analysis to map the network participation of each IC.

### Theory-of-mind task

The theory-of-mind task was performed by 20 subjects (12 females; mean age, 22.6 ± 0.8 years old). The study was approved by the Princeton University Institutional Review Board. All subjects gave informed written consent, had normal or corrected-to-normal vision, and had no history of psychiatric or neurological disorders.

We used a theory-of-mind localizer task from a study by Dodell-Feder et al. (2011), which contrasts brain activations during the attribution of beliefs to people (belief trials) with activations during judgments about the contents of photographs, maps, or signs (photo trials; Fig. 1*A*). The stimuli were provided by David Dodell-Feder, Nicholas Dufour, and Rebecca Saxe (http://saxelab.mit.edu/superloc.php). This task was chosen because of its extensive use in theory-of-mind studies and its popularity as a TPJ localizer task (Saxe and Powell, 2006; Mitchell, 2008; Scholz et al., 2009; Dodell-Feder et al., 2011). In each trial, the story was presented for 10 s, followed by a true/false question for 4 s and an intertrial interval (ITI) of 12 s. Participants responded to the questions using a button box. The task consisted of two runs of 10 trials each, and the order of stories was counterbalanced and equally distributed across the two runs. The BOLD response was modeled with a 14 s boxcar convolved with a standard hemodynamic response function (“waver” function; AFNI). A contrast between belief trials and photo trials is known to reveal activation in the TPJ and other theory-of-mind regions (Dodell-Feder et al., 2011). The reaction time was not significantly different in belief versus photo trials (mean ± SEM reaction time, 2.7 ± 0.10 and 2.6 ± 0.10 s, respectively; *p* = 0.18, paired *t* test).

### Episodic memory retrieval task

The episodic memory retrieval task was performed by 20 subjects, 1 of whom was excluded due to excessive movement (10 females; mean age, 22.3 ± 1.0 years). Subjects had normal or corrected-to-normal vision and no history of psychiatric or neurological disorders.

The episodic memory task was an old/new task based on a study by Konishi et al. (2000; Fig. 1*B*). The old/new paradigm was chosen because several studies have found TPJ activation with this contrast, and it has been part of meta-studies of functional overlap (Hutchinson et al., 2009, 2014; Cabeza et al., 2012). The task consisted of four runs. Each run comprised a memory-encoding phase and a memory-retrieval phase. In the encoding phase, subjects were asked to memorize 33 words, which were presented on the screen 1 word at a time (word duration, 2 s; trial duration, 3 s). In the retrieval phase, a randomized collection of words was presented in the same manner. These included 25 words from the learning phase and 50 new words. Twenty-five fixation trials were also randomly interspersed in each run. Subjects were asked to indicate with a button press whether or not they remembered the word as being from the learning run. There was no overlap of words among the four runs. The contrast between successfully retrieved old words (old trials) and successfully rejected new words (new trials) has been shown to evoke TPJ activation (Konishi et al., 2000). The word list was derived from the SUBTLEXus 1.00 word frequency database (Brysbaert and New, 2009) and consisted of five-letter words with a frequency of 5–10 per million. The BOLD response was modeled with a boxcar time course convolved with a standard hemodynamic response function (waver function; AFNI). The average accuracy did not differ between the old and new trials (mean ± SEM accuracy, 81.8 ± 2.7% and 87.9 ± 2.9%, respectively; *p* = 0.13, paired *t* test). The reaction time for new trials was slightly longer than that for old trials (mean ± SEM reaction time, 993 ± 27 and 927 ± 23 ms, respectively; *p* = 0.0013, paired *t* test).

### Social attribution of attention

The attribution-of-attention task was chosen because it was previously found to evoke robust activity in the TPJ even in individual subjects (Kelly et al., 2014) and offers a contrasting approach to testing social cognition from the belief attribution task. In the present study, we used the data collected in our previous study (Kelly et al., 2014) and reanalyzed it for the present study. The task (Fig. 1*C*) required subjects to rate the perceived level of awareness of a cartoon face (“Kevin”) for an object next to it. The direction of gaze of the face was manipulated (toward or away from the location of the object), and the emotional expression of the face either matched the valence of the object (e.g., a happy face paired with a cupcake or a frightened face paired with a house fire) or mismatched the valence of the object (e.g., a frightened face paired with a cupcake or a happy face paired with a house fire). Subjects rated Kevin’s level of awareness of the object on a scale of 1 (not aware) to 3 (very aware). When both the gaze and expression cues matched the object, subjects tended to rate Kevin as very aware (rating of 3). When both the gaze and expression cues mismatched the object, subjects tended to rate Kevin as unaware (rating of 1). When the cues to Kevin’s state of awareness were incompatible, with one cue suggesting awareness and the other suggesting unawareness, subjects tended to compromise between the two cues and rate Kevin’s awareness as intermediate (rating of 2). In the previous study, it was found that TPJ activity was significantly higher when subjects compromised between two incompatible cues to Kevin’s state of mind, presumably when the computation about Kevin’s state of mind was more difficult (“Hard trials”), and activity in the TPJ was significantly lower when the subjects used two compatible cues to Kevin’s state of mind, presumably when the computation about Kevin’s state of mind was easier (“Easy trials”).

The details of the paradigm are described in a previous publication (Kelly et al., 2014). Briefly, the behavioral task consisted of eight runs of 48 trials each. Each trial started with a fixation cross for 0.5 s, followed by a picture of an object for 1 s. The fixation cross returned for 0.5 s, and then was replaced by a cartoon face for 2 s. The object was presented either to the left or right and had either positive or negative valence. The gaze of the cartoon figure was either averted from or directed at the object, and the expression was either happy or alarmed. In this way, the gaze could either match (Gaze+) or mismatch (Gaze−) the location of the object, and the expression could either match (Expr+) or mismatch (Expr−) the valence of the object (see examples in Fig. 1*C*). Subjects were asked to indicate with one of three buttons whether the person was (1) not aware, (2) somewhat aware, or (3) very aware of the object. For the present study, we used data from the first 20 subjects of the total of 50 subjects from the previous study (Kelly et al., 2014; 8 females; mean age, 19.4 ± 0.4 years). The reason for analyzing a smaller subset of subjects was a limitation in memory on the compute cluster, which prevented a full 50-subject group level-ICA. Therefore, we chose to analyze the first 20 subjects in this task as an unbiased way of decreasing the computing requirements. We used the same regressors and contrast as in the previous study (described above), convolving the hard and easy trial types with a hemodynamic response function (waver function; AFNI) and testing the contrast hard versus easy.

### Attentional reorienting task

This task was performed by 20 subjects with normal or corrected-to-normal vision and no history of psychiatric or neurological disorders (11 females; mean age, 21.6 ± 0.6 years).

Attentional reorienting was tested using a Posner task modeled closely on a previous study (Mitchell, 2008; Fig. 1*D*). This task was chosen to represent reorienting to invalidly cued targets, which is thought to be a major function of the TPJ (Corbetta and Shulman, 2002; Corbetta et al., 2008; Mitchell, 2008; Geng and Vossel, 2013). The task consisted of five runs of 40 trials each. Subjects were given one practice run inside the scanner before the experiment started. The fixation screen consisted of a black background with a red central fixation plus sign (+) and two peripheral square boxes with white outlines. The peripheral boxes were centered 7° from the fixation and were 3° across. At the start of a trial, the central plus sign turned green for a fixation period of 700 ms and was replaced by a central cue consisting of an arrow for 800 ms. The arrow pointed either left or right, in a randomized counterbalanced order. After a pretarget period of 0.5–2 s, the target (a white asterisk) was presented in one of the two peripheral boxes for 100 ms. The posttarget time was selected to make the total trial duration 4 s, after which the fixation plus sign turned red again for a randomized ITI of 0.5–7.5 s. Subjects responded with a button press to indicate whether the target was on the left or on the right. The direction of the central cue predicted the side of the target in 75% of trials (valid trials) and was mismatched in 25% of trials (invalid trials). Subjects were informed that the arrow would predict the target in the majority of trials. A contrast between invalid and valid trials has been reported to reveal activity in the right TPJ and the ventral attention network (Corbetta et al., 2000). The BOLD response was modeled by convolving the stimulus timings with a gamma function. The reaction time for invalid trials was significantly longer than that for valid trials (mean ± SEM reaction time, 394 ± 16 and 362 ± 15 ms; *p* = 0.00007, paired *t* test).

### Oddball target detection task

This task was performed by 20 subjects, 1 of whom was excluded due to poor performance (10 females; mean age, 22.9 ± 1.0 years). The subjects had normal or corrected-to-normal vision and no history of psychiatric or neurological disorders.

The target detection task was a simple visual oddball task based on the study by Stevens et al. (2000; Fig. 1*E*). The oddball paradigm was chosen because it represents a nonspatial form of attentional reorienting that is often reported to cause TPJ activations (Corbetta et al., 2008; Cabeza et al., 2012). It consisted of four runs with 120 trials each. The standard stimulus was the letters “OOOO” presented centrally for 500 ms. The rare target stimulus consisted of the letters “XXXX” (4–7% of trials in each run) and was made task relevant by asking the subjects to report on how many targets they had seen after each run. A contrast between the target (target trials) and the standard stimuli (standard trials) was reported to evoke TPJ activity (Stevens et al., 2000). The BOLD response was modeled by convolving the stimulus timings with a gamma function.

### Distribution of subjects across the five tasks

For reasons of feasibility, every subject did not perform all tasks, which would have required a prohibitive number of hours and sessions for each subject. Instead, each task was analyzed separately. In effect, we performed five independent experiments. All statistical analyses were performed independently for each task, with no between-subjects statistical tests. The results are reported separately for each task and not used to draw conclusions about quantitative differences across tasks in the exact spatial location of ICs or the effect size of activity. In some cases, for tasks that required less run time, the same subjects participated in more than one task. Because of the separate analysis for each task, these overlaps in the subject pools are not of direct relevance to the analysis, but are nonetheless reported here. Fourteen subjects performed both the theory-of-mind task and the attentional reorienting task. Six subjects performed only the attentional reorienting task, and six subjects performed only the theory-of-mind task. Eighteen subjects performed both the episodic memory retrieval task and the target detection task. One subject performed only the episodic memory retrieval task, and one subject performed only the target detection task. For the social attribution-of-attention task, data from 20 subjects were used. In total across the five tasks, 66 subjects were tested.

### Magnetic resonance imaging

MRI images covering the whole cerebral cortex were acquired with a 20-channel receiver head coil on a Siemens Skyra scanner. Functional imaging used a gradient echo, echoplanar pulse sequence with a 64 × 64 matrix [27 axial slices; 4 mm thick; in-plane resolution, 3 × 3 mm; TR, 1.5 s; TE, 28 ms; flip angle (FA), 64°; generalized GRAPPA iPAT = 2. Anatomical imaging used an MP2RAGE sequence (256 × 240 matrix; TR, 5 s; TE, 2.98 ms; FA, 4°; 1 mm^2^ resolution; GRAPPA iPAT = 3). The reanalyzed data from the previous study (Kelly et al., 2014) were acquired on the same scanner. The functional data were acquired with a 64 × 64 matrix (35 axial slices; 3 mm thick; in-plane resolution, 3 × 3 mm; TR, 2 s; TE, 30 ms; FA, 77°), and the anatomical data were acquired with an MPRAGE sequence (256 × 224 matrix; TR, 2.3 s; FA, 9°; and with 1 mm^2^ [TE, 2.98 ms], 0.9 mm^2^ [TE, 3.08 ms], or 1.1 mm^2^ [TE, 2.93 ms] resolution).

### Preprocessing of fMRI data

Preprocessing was performed with AFNI (Cox, 1996) and FSL (Jenkinson et al., 2012). The functional data were slice time corrected and motion corrected with FSL (Jenkinson et al., 2002), and then detrended (linear and quadratic) with AFNI. The data were spatially normalized to the FSL MNI-152 template with AFNI, and spatially smoothed with a Gaussian kernel (5 mm FWHM). We used an ICA-based strategy for automatic removal of motion artifacts (ICA-AROMA; Pruim et al., 2015a). This toolbox runs single-session ICA with multivariate exploratory linear decomposition into independent components (MELODIC, FSL); and classifies motion-related ICs by assessing their high-frequency content, correlation with motion parameters, edge fraction, and cerebrospinal fluid fraction (Pruim et al., 2015a). ICA-AROMA removes noise ICs from the fMRI data by calling the FSL command fsl_regfilt (Beckmann and Smith, 2004). Such ICA-based denoising is effective in removing aberrant connectivity measures resulting from subject motion (Power et al., 2012; Salimi-Khorshidi et al., 2014; Pruim et al., 2015b).

### Group-level ICA

For each of the five tasks, the fMRI data were subjected to probabilistic ICA applied on temporally concatenated fMRI data, with all runs from all subjects concatenated into one matrix (MELODIC toolbox in FSL; Beckmann and Smith, 2004). We performed the ICA decomposition separately for each task (instead of grouping the tasks into one ICA) because we did not want to assume that the ICA decomposition would be the same across task conditions. We applied the ICA to the voxels within a region of interest (ROI) mask that included the TPJ and surrounding cortex to ensure that all relevant ICs were detected in their entirety. The mask was constructed from the standard surface cvs_avg35_inMNI152 in Freesurfer, using mri_label2vol to combine multiple labels from the aparc.a2009s atlas into one mask (G_pariet_inf-Supramar, G_pariet_inf-Angular, G_temp_sup-Plan_tempo, G_temp_sup-Lateral, G_temp_sup-G_T_transv, S_interm_prim-Jensen, S_temporal_sup, S_temporal_transverse) and trimming temporal cortex voxels anterior to the postcentral sulcus. This mask allowed a parcellation of the whole temporoparietal region. The reason for using localized ICA is that it allows a finer parcellation of the region (Sohn et al., 2012; Beissner et al., 2014; Igelström et al., 2015, 2016), but the exact extent of the mask is not critical for the results. The fMRI data were decomposed into 20 ICs, which isolates the major functional processes in the region (Igelström et al., 2015). ICs were thresholded at *z* = 2.3 for visual inspection (mixture-model threshold of *p* < 0.5) and at *z* = 4 for the creation of winner-take-all maps for the figures. Time courses for the figures were derived from the ICA mixing matrix (demeaned and variance-normalized signal; arbitrary *y*-axis; Beckmann and Smith, 2004). Event-related averaged waveforms were first calculated for each subject, and these were then averaged across subjects and presented as the mean ± SEM.

Task-related ICs were identified using a mixed-effects multiple regression (subjects as random effects) in R version 3.0.3 (nlme package version 3.1-113; Pinheiro et al., 2013; R Core Team, 2014), with the IC time courses as dependent variables and the predicted BOLD responses for each condition as independent variables (two trial types per task). The inclusion criteria for an IC to be accepted as task related were as follows: (1) a significant positive regression coefficient for the main condition of interest (“belief” trials in the theory-of-mind task; “old” trials in the episodic memory retrieval task; “hard” trials in the attribution-of-attention task; “invalid” trials in the attentional reorienting task; and “target” trials in the target detection task); and (2) a significant positive contrast in the general linear test (belief–photo; old–new; hard–easy; invalid–valid; target–standard). Any ICs located at the border of the mask outside the ROI were excluded (anterior superior temporal lobe, intraparietal sulcus, lateral fissure, postcentral sulcus, and anterior dorsal inferior parietal lobule).

In fMRI experiments, one possible concern is that eye movement might affect the measured cortical activity. The TPJ is not typically active in relation to eye movement, unlike more ventral regions in the superior temporal sulcus and more dorsal regions in the intraparietal sulcus. However, it is still important to ensure that the experimental design minimizes the possibility of an eye movement confound. In all five experimental designs, the analysis for identifying task-related ICs relied on the correlation of the IC time courses to trial-specific models of the BOLD response. Because the trial types were matched and counterbalanced with respect to visual features and processing demands (e.g., story/word length, left vs right targets), eye movements should not have influenced the identification of task-related ICs. All five tasks used a fixation point where necessary to stabilize eye position and were otherwise counterbalanced across the critical comparisons.

### Functional connectivity analysis

The CONN toolbox 15.c in SPM 12 (http://www.nitrc.org/projects/conn; Whitfield-Gabrieli and Nieto-Castanon, 2012) was used for seed-to-voxel connectivity analysis (Biswal et al., 1995) using the subject-specific IC time courses as seed time courses. Conventional bivariate correlation analysis was used, with a voxelwise threshold of *p* < 0.001 uncorrected and a cluster extent threshold of *p* < 0.05 (false discovery rate corrected).

## Results

We present the results from each of the five tasks separately and then discuss the comparison among the tasks.

### Theory of mind

**Figure 2. F2:**
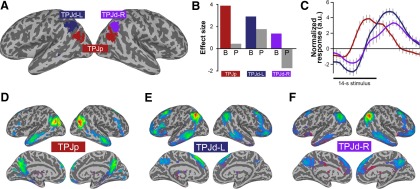
ICs activated in the theory-of-mind task. ***A***, Location of significant ICs shown as a winner-take-all map, created from *z*-score maps thresholded at *z* > 4. ***B***, Regression coefficients for the belief (B) and photo (P) conditions for the three significantly task-related ICs. ***C***, Event-related IC time courses for the belief condition during the 14 s story-plus-question block (black bar) for TPJp (red), TPJd-L (blue), and TPJd-R (purple). The *y*-axis is shown in arbitrary units. ***D–F***, Connectivity patterns of the three task-related ICs obtained in the theory-of-mind task, as follows: TPJp (***D***), TPJd-L (***E***), and TPJd-R (***F***).

The theory-of-mind task activated a bilateral posterior IC resembling TPJp in our previous studies (Fig. 2*A*, red). It also activated two lateralized dorsal components, in the region of the right and left TPJd (Fig. 2*A*, TPJd-R, purple, TPJd-L, blue). The TPJp activity was specific for the belief trials, with a large effect for the belief (“B”) condition (Fig. 2*B*, red bar), and no significant activity related to the photo (“P”) condition (Fig. 2*B*, gray bar). The activation patterns for TPJd-R and TPJd-L were distinct from that of TPJp, with lower specificity for the belief condition. TPJd-L showed some activity in photo trials, whereas the activity of TPJd-R was negatively related to photo trials (Fig. 2*B*, gray bars).

We extracted belief-related BOLD time courses from the three task-related ICs to examine the temporal properties of these processes. We found a latency difference between the posterior and dorsal components. While TPJp showed a typical BOLD response during the story stimulus (peak, 11 s), TPJd-R and TPJd-L showed a later onset and peak (peaks, 17-18 s), indicating involvement in a more delayed process compared with that of TPJp (Fig. 2*C*).

To further characterize these zones of activation and relate them to previously reported TPJ subdivisions (Mars et al., 2012; Bzdok et al., 2013; Igelström et al., 2015), we performed a functional connectivity analysis using the IC time courses as seed time courses. The most strongly and selectively recruited IC in the theory-of-mind task, TPJp, was connected with default-mode network regions, including precuneus, STS, and medial and lateral PFC (Fig. 2*D*). This connectivity is similar to that of TPJp reported by others and us (Mars et al., 2012; Bzdok et al., 2013; Igelström et al., 2015), and similar to regions activated by theory-of-mind tasks (Dodell-Feder et al., 2011). TPJd-L and TPJd-R were connected with lateralized frontoparietal networks involving the inferior temporal lobe, lateral and superior frontal cortex, and precuneus (Fig. 2*E*,*F*). These connectivity patterns also agree with the network participation of TPJd reported previously (Mars et al., 2012, termed “IPL”; Igelström et al., 2015).

### Episodic memory retrieval

**Figure 3. F3:**
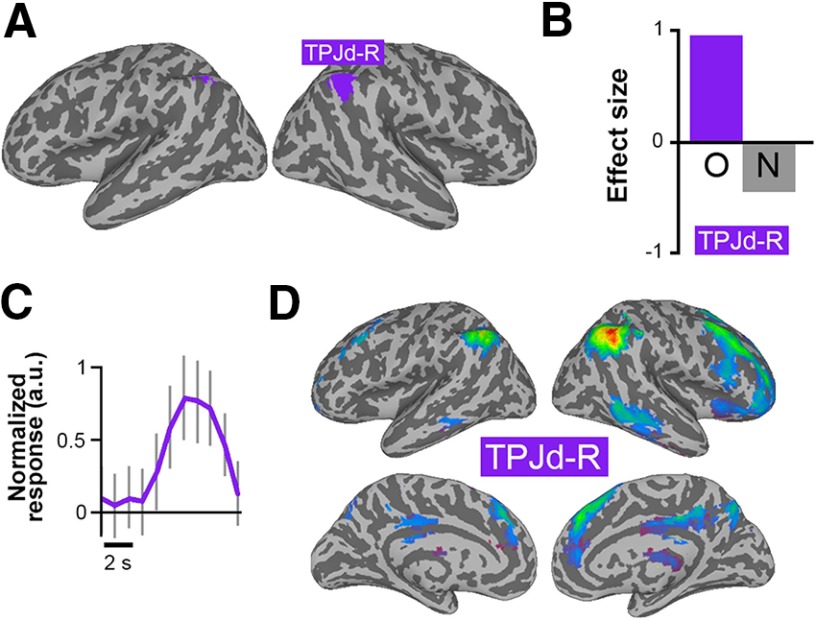
ICs activated during episodic memory retrieval. ***A***, Location of the significant IC created from a *z*-score map thresholded at *z* > 4. ***B***, Regression coefficients for the old (O) and new (N) conditions for the significantly task-related IC. ***C***, Event-related IC time course for the old condition for TPJd-R. The *y*-axis is shown in arbitrary units. ***D***, Connectivity of TPJd-R.

The episodic memory task activated an IC in the right angular gyrus, which was also located in the region of TPJd-R (Fig. 3*A*). Its activity was specific for memory retrieval (Fig. 3*B*), and it showed an activity peak at 7 s after stimulus onset (Fig. 3*C*). The IC was connected to the same right-lateralized frontoparietal network as the TPJd-R component activated by the theory-of-mind task (compare Figs. 3*D*, 2*F*).

There was a left-lateralized component in a similar region that did not reach our statistical criteria. This IC, in the area of the TPJd-L, showed a significant old/new contrast, but it did not pass the statistical threshold for the old condition (β = 0.50, *p* = 0.026). Therefore, we did not include it here as a significantly activated IC.

### Attribution of attention

**Figure 4. F4:**
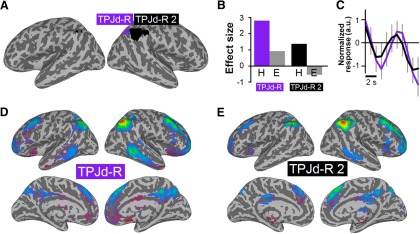
ICs activated during social attribution of attention. ***A***, Location of significant ICs shown as a winner-take-all map, created from *z*-score maps thresholded at *z* > 4. ***B***, Regression coefficients for the hard (H) and easy (E) conditions for the two significantly task-related ICs. ***C***, Event-related IC time courses for the hard condition for TPJd-R (purple) and TPJd-R2 (black). Due to the rapid trial presentation in this task, the signal was still returning to baseline at the beginning of the trial. The *y*-axis is shown in arbitrary units. ***D***, ***E***, Connectivity of the task-related ICs, as follows: TPJd-R (***D***) and TPJd-R 2 (***E***).

The attribution-of-attention task activated two spatially similar ICs in the region around the TPJd-R (Fig. 4*A*, TPJd-R, purple, TPJd-R 2, black). TPJd-R was more strongly activated than TPJd-R 2 (Fig. 4*B*), but both were connected to the same right frontoparietal network (Fig. 4*D*,*E*). The event-related time courses showed no difference in latency or shape, suggesting that these processes reflected erroneous splitting of a single process. The frontoparietal network was similar to the networks connected with TPJd-R in the theory-of-mind and episodic memory retrieval tasks.[Fig F2][Fig F3][Fig F4] Figure 4*C* shows the time courses of TPJd-R and TPJd-R 2 (peaks, 7 s). Because of the rapid presentation of stimuli in this task, the time course showed activity from the previous trial dropping, and then rising again in response to the current trial.

### Attentional reorienting

**Figure 5.**
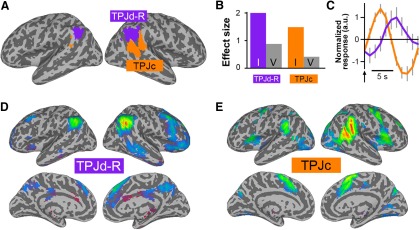
ICs activated during the attentional reorienting task. ***A***, Location of significant ICs shown as a winner-take-all map, created from *z*-score maps thresholded at *z* > 4. ***B***, Regression coefficients for the invalid (I) and valid (V) conditions for the two significantly task-related ICs. ***C***, Event-related IC time courses for invalid trials for the TPJd-R (purple) and TPJc (orange). The *y*-axis is shown in arbitrary units and the arrow shows stimulus onset. ***D***, ***E***, Connectivity of the task-related ICs, as follows: TPJd-R (***D***) and TPJc (***E***).

The attentional reorienting task showed two significant ICs (Fig. 5*A*,*B*). One was located in the region of the TPJd-R, and the other was located anterior to TPJd-R in a location close to TPJc reported previously (Igelström et al., 2015). Event-related BOLD time courses for TPJd-R and TPJc again showed a long-latency response in TPJd-R (peak, 7 s) compared with TPJc (peak, 4 s; Fig. 5*C*).

The TPJd-R component was connected to the right frontoparietal control network (Fig. 5*D*). TPJc was connected with regions in the ventral attention network, including the anterior cingulate cortex, anterior insula, and the inferior frontal cortex, and showed a strong bias toward the right hemisphere (Fig. 5*E*).

### Target detection

**Figure 6.**
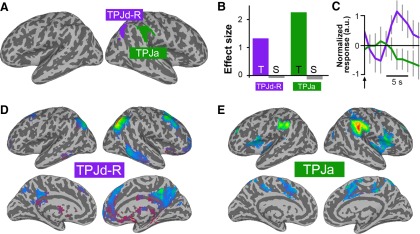
ICs activated in the oddball target detection task. ***A***, Location of significant ICs shown as a winner-take-all map, created from *z*-score maps thresholded at *z* > 4. ***B***, Regression coefficients for the target (T) and standard (S) conditions for the two significantly task-related ICs. ***C***, Event-related IC time courses for the target condition for the TPJd-R (purple) and TPJa (green). The *y*-axis is shown in arbitrary units and the arrow shows stimulus onset. ***D***, ***E***, Connectivity of the task-related ICs, as follows: TPJd-R (***D***) and TPJa (***E***).

The target detection task activated one IC matching the TPJd-R and one IC in the anterior supramarginal gyrus in the region labeled TPJa in our previous study (Fig. 6*A*,*B*). The location of TPJd-R appeared to be located more posterior than in the other tasks (Fig. 6*A*); however, it was connected with the same right frontoparietal network as the TPJd-R regions activated in the other tasks and in our previous study (Fig. 6*D*). TPJa was connected to regions of the ventral attention network, including the right inferior frontal gyrus, anterior insula, and anterior cingulate cortex (Fig. 6*E*). As in the theory-of-mind and attentional reorienting tasks, the event-related BOLD time course of TPJd-R showed a longer-latency response (peak, 7 s) compared with the early peak of TPJa (peak, 4 s; Fig. 6*C*).

### Summary

The attentional reorienting and target detection tasks activated supramarginal regions (TPJc and TPJa, respectively) connected with the ventral attention network, whereas the theory-of-mind task uniquely activated the TPJp, which was connected with the default-mode network. All five tasks activated TPJd-R in the dorsal angular gyrus, which showed connectivity with the right-lateralized frontoparietal control network. The BOLD response of TPJd-R showed a longer latency than that of the other ICs activated in the same task.

## Discussion

**Figure 7.**
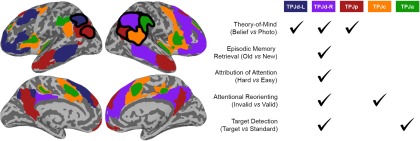
Simplified schematic summary of task activations. The task-related temporoparietal independent components from Figures 2–6 are shown in their approximate locations with black outlines, and their network connectivity is marked with matching colors but no outlines (see Figures 2-6 for the exact distribution and connectivity of the independent components). All tasks activated regions within the TPJd-R (purple area), which was connected with the right-lateralized frontoparietal network. The theory-of-mind task also activated TPJd-L (blue; connected with the left-lateralized frontoparietal network) and TPJp (red; connected with the default-mode network). The attentional reorienting task and the target detection task activated TPJc and TPJa, respectively (orange and green), which were connected with partially overlapping regions of the ventral attention network.

The present findings show that the right dorsal TPJ is active during a range of tasks, including social, attentional, and memory tasks, whereas other TPJ zones are active in a more task-specific way (summarized in Fig. 7). The domain-specific TPJ processes in TPJp, TPJc, and TPJa showed a shorter-latency BOLD response compared with the domain-general process in TPJd, further strengthening the suggestion that the computations performed by these areas are distinct from each other.

### Separation of functions within the TPJ

There was clear spatial separation of the theory-of-mind task (activating the TPJp), and the attentional reorienting and target detection tasks (activating the TPJc and TPJa, respectively). These TPJ regions were also connected to distinct networks. The TPJp was connected with classic theory-of-mind/default-mode regions, including precuneus and medial prefrontal cortex. The TPJc and TPJa were both connected with the ventral attention network. Thus, this study shows spatial separation of processes related to theory-of-mind and attentional reorienting. The spatial relationship between theory-of-mind and attentional functions has been debated in previous studies, but a general pattern of a more posterior locus for theory of mind has emerged (Decety and Lamm, 2007; Carter and Huettel, 2013; Geng and Vossel, 2013; Kubit and Jack, 2013). The clear separation observed here is likely made possible by linear decomposition of overlapping signals using ICA. The difference between TPJc and TPJa was more ambiguous, especially given their similar connectivity patterns. Attentional reorienting tasks and target detection tasks have generally been grouped together in meta-studies, but, when they were separated, a more anterior location of target detection activity compared with activity in attentional reorienting tasks was seen (Kubit and Jack, 2013), which is similar to the present findings.

The dorsal location of activity in episodic memory retrieval in the present experiment was similar to the dorsal activations seen in previous studies (Konishi et al., 2000; Wagner et al., 2005; Hutchinson et al., 2009). However, episodic memory retrieval activity in old/new tasks is often dominantly expressed in the left hemisphere, in contrast to the right lateralization observed here. Our result does not exclude the possibility of relevant left-lateralized retrieval-related activity. First, the TPJd-R component showed strong functional connectivity to the left TPJd (Fig. 3*C*), indicating involvement of the opposite hemisphere. Second, we observed a TPJd-L component that did not meet our strict inclusion criteria of a significant association with both the old condition and the old/new contrast. In a conventional analysis aiming to identify activity significant for the old/new contrast, regardless of the significance of the old trials, TPJd-L would have been identified as active.

### A global role of the dorsal TPJ

The TPJ has been suggested to be a hub or nexus in which multiple brain systems converge and communicate (Carter and Huettel, 2013; Geng and Vossel, 2013). In the current study, not the whole TPJ, but specifically the TPJd-R, the subdivision connected to the right frontoparietal control network, was recruited in all five tasks. These findings suggest that the TPJd may be a major site of functional convergence and interaction.

An influential theory about TPJ function suggests that it is involved in postperceptual processes, such as the updating of internal models of the current context based on incoming sensory information (Geng and Vossel, 2013). It was suggested that stimuli that violate expectations in some way, such as invalidly cued targets, oddballs, or conflicting social cues, activate the TPJ for this reason (Geng and Vossel, 2013). In the memory domain, the TPJ has been suggested to be a buffer or convergence zone to manipulate or bind episodic memories (Baddeley, 2000; Vilberg and Rugg, 2008; Shimamura, 2011). An integrative function of the TPJ is also consistent with roles of the TPJ in representing the subjective experience of one’s own body (Tsakiris et al., 2008; Blanke et al., 2015) or one’s own state of awareness (Graziano and Kastner, 2011; Kelly et al., 2014), functions that also rely on the integration of external stimuli with internal models. The TPJd is part of the frontoparietal control system, which is spatially interposed between the dorsal attention network and the default-mode network (Vincent et al., 2008) and serves a regulatory role in maintaining a balance between them (Spreng et al., 2013). This frontoparietal system has been suggested to be a “flexible hub” that rapidly adapts its brain-wide connectivity according to the current context and task demands (Addis et al., 2007; Christoff et al., 2009; Summerfield et al., 2010; Gerlach et al., 2011; Ellamil et al., 2012; Meyer et al., 2012; Cole et al., 2013). The TPJd is thus positioned to play a central role in multiple interacting brain systems.

### Limitations of findings

An important limitation of this study is that it did not quantify the voxelwise differences across the five tasks in the spatial location of the TPJ ICs. Our data-driven method was effective in reducing TPJ fMRI data to the main spatiotemporal processes in the region, which allowed us to identify task-related activity with unprecedented power and clean separation. However, the TPJ heterogeneity and random starting parameters of the ICA algorithm make the method unsuitable for quantifying and comparing the exact coordinates of activity. The exact spatial configuration of group-level parcellations can differ for each subject cohort, as observed in our previous study (Igelström et al., 2015). Therefore, it is not known whether the slight spatial variability between the tasks was caused by anatomical heterogeneity, task-related activity, or variability of the ICA algorithm. For example, the heterogeneity observed in the localization of TPJd-R among different tasks may indicate that slightly different TPJd regions interact with the frontoparietal control network in different tasks, may reflect anatomical differences in TPJ organization among subject cohorts, and may reflect the random starting parameters of the ICA.

### Conclusions

We used localized ICA to study the TPJ during five behavioral tasks known to activate the region. Localized ICA can isolate spatiotemporal processes from each other and from the background noise, distilling the noisy fMRI data to a small number of processes and minimizing multiple comparisons. The local ICs can then be placed into brain-wide networks using functional connectivity analysis. If two ICs in different subject cohorts show similar connectivity, they are likely to be, if not functionally equivalent, at least very closely related. Using this method, we found that the TPJ contains both domain-specific and domain-general neural processes, which are separable in space and show distinct temporal properties. Processes specific to attentional reorienting and target detection were located in the supramarginal gyrus, and were associated with the ventral attention network. A posterior TPJ component specifically contained theory-of-mind activity and was connected with default-mode regions. A right-lateralized dorsal TPJ zone within the frontoparietal control network was activated across all the tested domains and showed a longer-latency BOLD response compared with the domain-specific processes. These findings strongly support the concept of the TPJ as a cognitive hub that mediates interactions among multiple brain networks, but also show that more functionally specific processes occur adjacent to the zone of convergence.

*Note added in Proof* - The last name of 4th author was accidentally left off this article that was published on-line April 12, 2016, as an Early Release. The author line has since been corrected.

## Synthesis

The decision was a result of the Reviewing Editor Philippe Tobler and the peer reviewers coming together and discussing their recommendations until a consensus was reached. A fact-based synthesis statement explaining their decision and outlining what is needed to prepare a revision is listed below. The following reviewer(s) agreed to reveal their identity: Scott Huettel

Dear Dr. Igelstrom,

Your revised paper has now been seen by two reviewers. While they still have concerns about the study (see below for your information), they recommended to accept.

"This study provides evidence for the heterogeneity of functions supported by the TPJ using a combination of traditional functional MRI methods and functional connectivity analyses. One of the reviewers still believes that the use of separate but overlapping samples poses interpretative challenges for the spatial organization of results. Yes, there are aspects of their analyses that partially ameliorate those challenges (e.g., measuring connectivity). But even those analyses differ dramatically between seeds that differ only slightly in location -- which is exactly what is found for TPJ. In short, I still find this to be at best an imperfect dataset for establishing the functional organization of TPJ. Yet, even if this isn't close to the final word on TPJ organization, it is an important step and still contributes new information. So, I am hopeful that it inspires the authors (or others) to repeat these analyses in other datasets that could provide additional, cleaner answers."
